# Supplementary material for: A Cre-dependent massively parallel reporter assay allows for cell-type specific assessment of the functional effects of non-coding elements in vivo
Source: Commun Biol. 2023 Nov 13;6:1151. doi: 10.1038/s42003-023-05483-w (PMC10641075; doi:10.1038/s42003-023-05483-w)
Supplement: Supplementary file 1 — Supplementary Information [file 42003_2023_5483_MOESM1_ESM.pdf]

Supplementary Information

Supplementary Figures

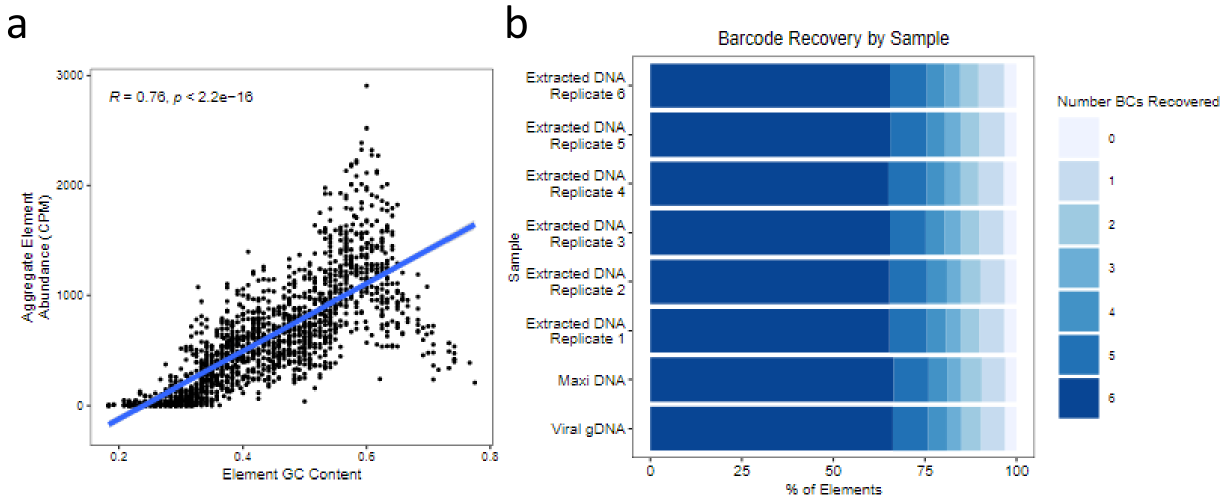

Supplementary Figure 1. Library quality control

a) Average element abundance vs element GC content b) Barcode recovery by sample. n=6 biological replicates, summed.

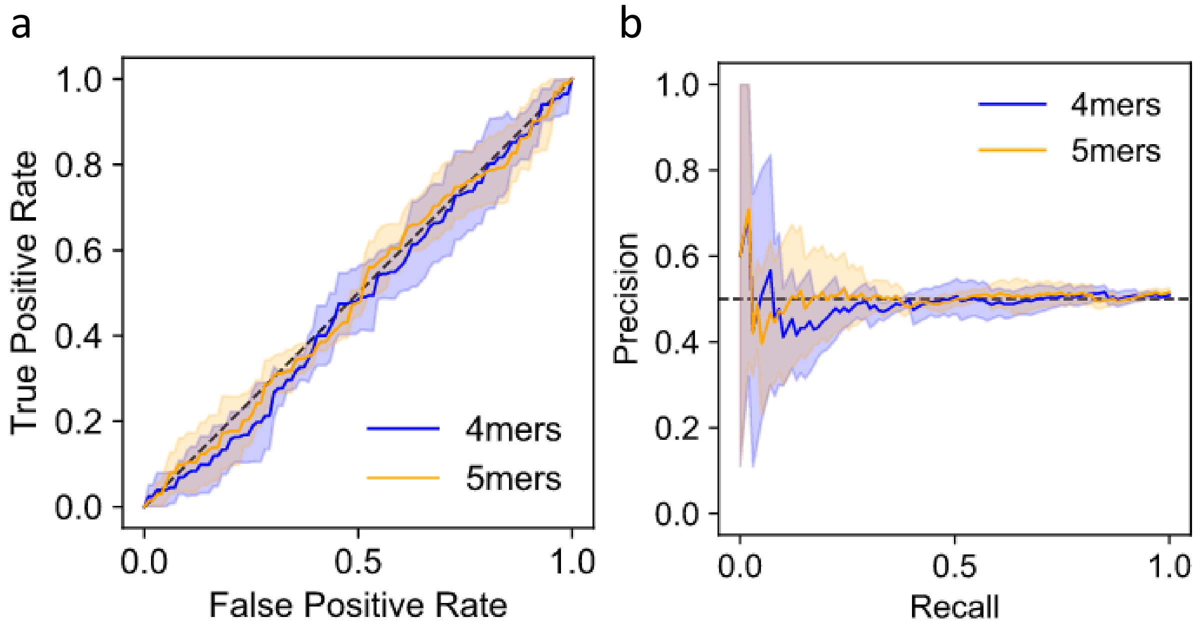

Supplementary Figure 2. SVM data from random labels

a) ROC and b)PRC for k-mer SVMs to classify high and low expressing shuffled elements. Shaded area represents 1 standard deviation based on five-fold cross-validation.

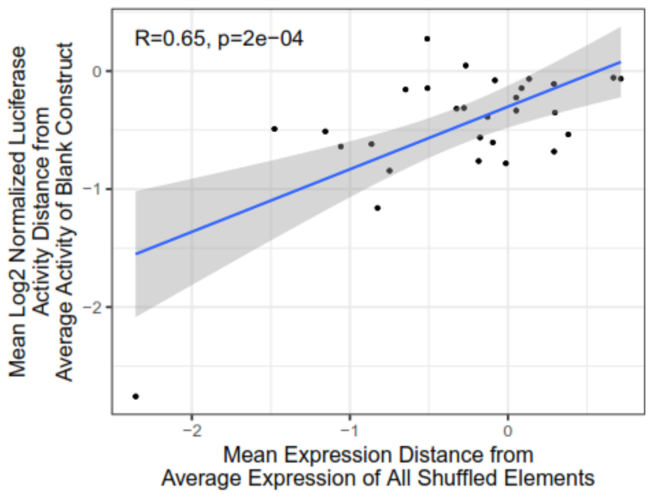

Supplementary Figure 3. Comparison of element effects on RNA levels to protein production. 28 elements from the MPRA library were cloned into a luciferase reporter plasmid in the 3'UTR position and protein production was assessed. Each sample was normalized to a blank construct (no inserted 3'UTR) vector to identify elements that increased (positive numbers) or decreased (negative numbers) compared to control (Y-axis). These are compared to the corresponding RNA results from the MPRA, normalizing each element to the mean of all shuffled controls to those that relatively increased (positive numbers) or decreased (negative numbers) DNA-normalized RNA levels (X-axis). n=3 biological replicates, averaged for each point. Shaded region indicates 95% confidence interval around linear regression line.

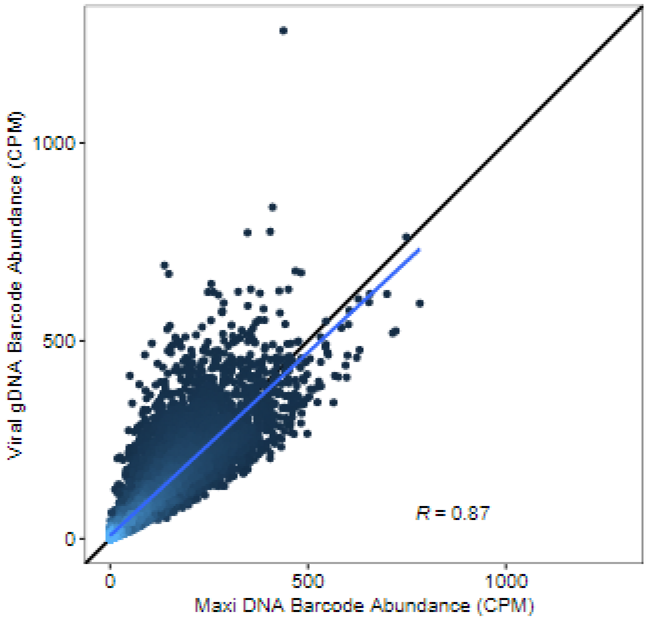

Supplementary Figure 4. Viral packaging correlates with plasmid DNA barcode counts

Scatter plot showing correlation between maxiprep plasmid DNA and viral DNA barcode counts. n=3 replicate library creations.

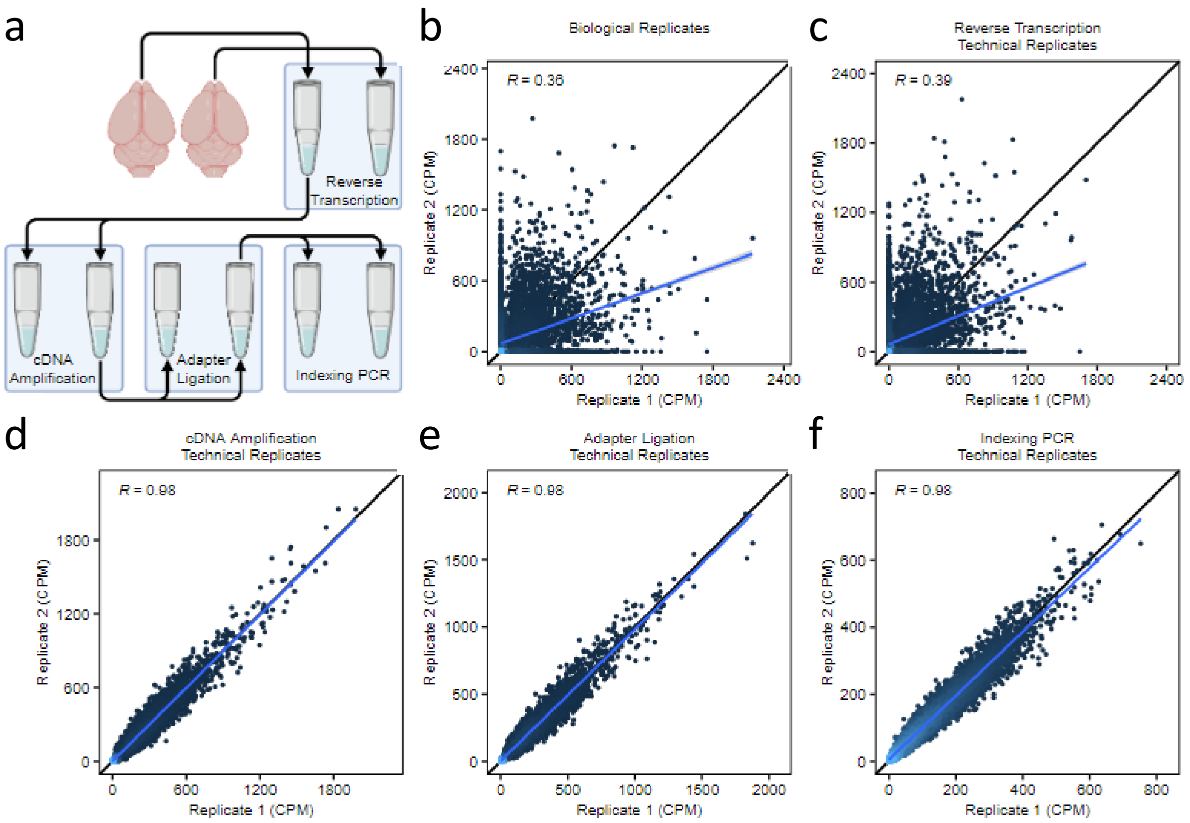

Supplementary Figure 5: Reaction splitting to determine source of jackpotting

a) Various steps of the MPRA library preparation pipeline. Brains are harvested and extracted total RNA reverse transcribed to create cDNA, cDNA is amplified, adapters are ligated on, and sequencing indexes are added to complete libraries. Panel was created with Biorender.com. b) Representative scatter plot showing correlation of biological replicates from Rbp4/Vglut animals. c) Library correlation of technical replicates when splitting at the cDNA synthesis stage. d) Library correlation of technical replicates when splitting at the cDNA amplification stage. e) Library correlation of technical replicates when splitting at the adapter ligation stage. f) Library correlation of technical replicates when splitting at the indexing PCR stage. Replicates are indicated for all graphs.
