# Supplementary material for: A Cre-dependent massively parallel reporter assay allows for cell-type specific assessment of the functional effects of non-coding elements in vivo
Source: Commun Biol. 2023 Nov 13;6:1151. doi: 10.1038/s42003-023-05483-w (PMC10641075; doi:10.1038/s42003-023-05483-w)
Supplement: Supplementary file 2 — Description of Additional Supplementary Files [file 42003_2023_5483_MOESM2_ESM.pdf]

Description of Additional Supplementary Files

File name: Supplementary Data 1
Description: List of variants used for MPRA library, and corresponding transcripts.

File name: Supplementary Data 2
Description: Overview of replicates, sequencing statistics, and results for all MPRA experiments.

File name: Supplementary Data 3
Description: Analysis of motif enrichment in highest, lowest, highest pyramidal neuron, and highest medium spiny neuron expressed elements.

File name: Supplementary Data 4
Description: Log2 Fold-Change of Reference vs. Shuffled Elements.

File name: Supplementary Data 5
Description: Log2 Fold-Change of Reference vs. Variant Elements.

File name: Supplementary Data 6
Description: Oligonucleotide sequences included in the MPRA library and their corresponding barcodes.

File name: Supplementary Data 7
Description: Raw data used to produce Figures 2b, 3e, and 3i.
