# Supplementary material for: A Cre-dependent massively parallel reporter assay allows for cell-type specific assessment of the functional effects of non-coding elements in vivo
Source: Commun Biol. 2023 Nov 13;6:1151. doi: 10.1038/s42003-023-05483-w (PMC10641075; doi:10.1038/s42003-023-05483-w)
Supplement: Supplementary file 10 — Reporting Summary [file 42003_2023_5483_MOESM10_ESM.pdf]

nature portfolio

Corresponding author(s): Joseph Dougherty

Last updated by author(s): 8/22/2023

Reporting Summary

Nature Portfolio wishes to improve the reproducibility of the work that we publish. This form provides structure for consistency and transparency in reporting. For further information on Nature Portfolio policies, see our Editorial Policies Editorial Policy Checklist

Statistics

For all statistical analyses, confirm that the following items are present in the figure legend, table legend, main text, or Methods section.

n/a	Confirmed	
☐	☒	The exact sample size (*n*
☐	☒	A statement on whether measurements were taken from distinct samples or whether the same sample was measured repeatedly
☐	☒	The statistical test(s) used AND whether they are one- or two-sided<br>*Only common tests should be described solely by name; describe more complex techniques in the Methods section.*
☒	☐	A description of all covariates tested
☐	☒	A description of any assumptions or corrections, such as tests of normality and adjustment for multiple comparisons
☐	☒	A full description of the statistical parameters including central tendency (e.g. means) or other basic estimates (e.g. regression coefficient) AND variation (e.g. standard deviation) or associated estimates of uncertainty (e.g. confidence intervals)
☐	☒	For null hypothesis testing, the test statistic (e.g. *F t r P*<br>*Give P values as exact values whenever suitable.*
☒	☐	For Bayesian analysis, information on the choice of priors and Markov chain Monte Carlo settings
☒	☐	For hierarchical and complex designs, identification of the appropriate level for tests and full reporting of outcomes
☐	☒	Estimates of effect sizes (e.g. Cohen's *d r*

Our web collection on statistics for biologists

Software and code

Policy information about availability of computer code

Data collection	Code is available at bitbucket: https://bitbucket.org/jdlabteam/mpra_lib_1.0_methods_paper/src/master/
Data analysis	Code is available at bitbucket: https://bitbucket.org/jdlabteam/mpra_lib_1.0_methods_paper/src/master/

For manuscripts utilizing custom algorithms or software that are central to the research but not yet described in published literature, software must be made available to editors and reviewers. We strongly encourage code deposition in a community repository (e.g. GitHub). See the Nature Portfolio guidelines for submitting code & software

Data

Policy information about availability of data

All manuscripts must include a data availability statement

- Accession codes, unique identifiers, or web links for publicly available datasets
- A description of any restrictions on data availability
- For clinical datasets or third party data, please ensure that the statement adheres to our policy

MPRA libraries are available upon request. MPRA data are deposited with GEO at GSE186455.

Field-specific reporting

Please select the one below that is the best fit for your research. If you are not sure, read the appropriate sections before making your selection.

☒ Life sciences ☐ Behavioural & social sciences ☐ Ecological, evolutionary & environmental sciences

For a reference copy of the document with all sections, see nature.com/documents/nr-reporting-summary-flat.pdf

Life sciences study design

All studies must disclose on these points even when the disclosure is negative.

Sample size	Each MPRA was done on at least six biological samples. Refer to Supplemental Table 2 for details.
Data exclusions	Elements with less than three barcodes represented were excluded. Refer to Methods section.
Replication	MPRA experiments were done on at least six biological samples. 28 elements were taken for further validation in a luciferase assay.
Randomization	All animals were given the same library. N/A
Blinding	All animals were in same group. N/A

Reporting for specific materials, systems and methods

We require information from authors about some types of materials, experimental systems and methods used in many studies. Here, indicate whether each material, system or method listed is relevant to your study. If you are not sure if a list item applies to your research, read the appropriate section before selecting a response.

Materials & experimental systems

n/a	Involved in the study	
☐	☒	Antibodies
☐	☒	Eukaryotic cell lines
☒	☐	Palaeontology and archaeology
☐	☒	Animals and other organisms
☒	☐	Clinical data
☒	☐	Dual use research of concern
☒	☐	Plants

Methods

n/a	Involved in the study	
☒	☐	ChIP-seq
☒	☐	Flow cytometry
☒	☐	MRI-based neuroimaging

Antibodies

Antibodies used	anti-RFP, Rabbit Polyclonal, Rockland #600-401-379<br>anti-GFAP, Goat polyclonal, Abcam #ab53554<br>anti-CNPase, Mouse monoclocal IgG1 clone 11-5B, Millipore-Sigma #MAB326<br>Alexa Fluor 488 Conjugated Anti-Goat IgG, Donkey Polyclonal, Jackson Immunoresearch #705-545-147<br>Alexa Fluor 647 Conjugated Anti-Mouse IgG1, Goat<br>Alexa Fluor 568 Conjugated Anti-Rabbit IgG, Donkey Polyclonal, Thermofisher #A10042
Validation	Validation information can be found on manufacturers' websites.

Eukaryotic cell lines

Policy information about cell lines and Sex and Gender in Research

Cell line source(s)	Neuro-2a, ATCC #CCL-131
Authentication	Cell Lines were authenticated by manufacturer.
Mycoplasma contamination	Cell line tested negative for mycoplasma contamination.
Commonly misidentified lines (See ICLAC	N/A

Animals and other research organisms

Policy information about studies involving animals ; ARRIVE guidelines Sex and Gender in Research

Laboratory animals	Mus musculus<br>Jackson Laboratory Strain #023527, B6;129S-Slc17a7tm1.1(cre)Hze/J (Vglut1-IRES2-Cre-D)<br>Jackson Laboratory Strain #016962, Slc32a1tm2(cre)Lowl/J (Vgat-IRES-Cre)<br>Tg(RBP4-cre)KL100Gsat/Mmcd (RRID:MMRRC_037128-UCD; Beltramo et al., 2013)
Wild animals	N/A
Reporting on sex	Both sexes were used. Did not segregate by sex as mice were prepubescent.
Field-collected samples	N/A
Ethics oversight	All procedures involving animals were approved by the Institutional Animal Care and Use Committee (IACUC) at Washington University in St. Louis, MO

Note that full information on the approval of the study protocol must also be provided in the manuscript.

Dual use research of concern

Policy information about dual use research of concern

Hazards

Could the accidental, deliberate or reckless misuse of agents or technologies generated in the work, or the application of information presented in the manuscript, pose a threat to:

No	Yes	
☒	☐	Public health
☒	☐	National security
☒	☐	Crops and/or livestock
☒	☐	Ecosystems
☒	☐	Any other significant area

Experiments of concern

Does the work involve any of these experiments of concern:

No	Yes	
☒	☐	Demonstrate how to render a vaccine ineffective
☒	☐	Confer resistance to therapeutically useful antibiotics or antiviral agents
☒	☐	Enhance the virulence of a pathogen or render a nonpathogen virulent
☒	☐	Increase transmissibility of a pathogen
☒	☐	Alter the host range of a pathogen
☒	☐	Enable evasion of diagnostic/detection modalities
☒	☐	Enable the weaponization of a biological agent or toxin
☒	☐	Any other potentially harmful combination of experiments and agents
